# Mitochondrial Network State Scales mtDNA Genetic Dynamics

**DOI:** 10.1534/genetics.119.302423

**Published:** 2019-06-28

**Authors:** Juvid Aryaman, Charlotte Bowles, Nick S. Jones, Iain G. Johnston

**Affiliations:** * Department of Mathematics, Imperial College London, SW7 2AZ, United Kingdom; † Department of Clinical Neurosciences, University of Cambridge, CB2 0QQ, United Kingdom; ‡ Medical Research Council Mitochondrial Biology Unit, University of Cambridge, CB2 0XY, United Kingdom; § School of Biosciences, University of Birmingham, B15 2TT, United Kingdom; ** Engineering and Physical Sciences Research Council Centre for the Mathematics of Precision Healthcare, Imperial College London, SW7 2AZ, United Kingdom; †† Faculty of Mathematics and Natural Sciences, University of Bergen, 5007, Norway; ‡‡ Alan Turing Institute, London NW1 2DB, United Kingdom

**Keywords:** mitochondrial DNA, mitochondrial networks, heteroplasmy variance, cellular noise

## Abstract

Mitochondrial DNA (mtDNA) mutations cause severe congenital diseases but may also be associated with healthy aging. mtDNA is stochastically replicated and degraded, and exists within organelles which undergo dynamic fusion and fission. The role of the resulting mitochondrial networks in the time evolution of the cellular proportion of mutated mtDNA molecules (heteroplasmy), and cell-to-cell variability in heteroplasmy (heteroplasmy variance), remains incompletely understood. Heteroplasmy variance is particularly important since it modulates the number of pathological cells in a tissue. Here, we provide the first wide-reaching theoretical framework which bridges mitochondrial network and genetic states. We show that, under a range of conditions, the (genetic) rate of increase in heteroplasmy variance and *de novo* mutation are proportionally modulated by the (physical) fraction of unfused mitochondria, independently of the absolute fission–fusion rate. In the context of selective fusion, we show that intermediate fusion:fission ratios are optimal for the clearance of mtDNA mutants. Our findings imply that modulating network state, mitophagy rate, and copy number to slow down heteroplasmy dynamics when mean heteroplasmy is low could have therapeutic advantages for mitochondrial disease and healthy aging.

MITOCHONDRIAL DNA (mtDNA) encodes elements of the respiratory system vital for cellular function. Mutation of mtDNA is one of several leading hypotheses for the cause of normal aging (López-Otín *et al.* 2013; Kauppila *et al.* 2017), as well as underlying a number of heritable mtDNA-related diseases (Schon *et al.* 2012). Cells typically contain hundreds, or thousands, of copies of mtDNA per cell: each molecule encodes crucial components of the electron transport chain, which generates energy for the cell in the form of ATP. Consequently, the mitochondrial phenotype of a single cell is determined, in part, by its fluctuating population of mtDNA molecules (Wallace and Chalkia 2013; Stewart and Chinnery 2015; Aryaman *et al.* 2019; Johnston 2019). The broad biomedical implications of mtDNA mutation, combined with the countable nature of mtDNAs and the stochastic nature of their dynamics, offer the opportunity for mathematical understanding to provide important insights into human health and disease (Aryaman *et al.* 2019).

An important observation in mitochondrial physiology is the threshold effect, whereby cells may often tolerate relatively high levels of mtDNA mutation until the fraction of mutated mtDNAs (termed heteroplasmy) exceeds a certain critical value where a pathological phenotype occurs (Rossignol *et al.* 2003; Picard *et al.* 2014; Stewart and Chinnery 2015; Aryaman *et al.* 2017). Fluctuations within individual cells mean that the fraction of mutant mtDNAs per cell is not constant within a tissue (Figure 1A), but follows a probability distribution which changes with time (Figure 1B). Here, motivated by a general picture of aging, we will largely focus on the setting of nondividing cells, which possess two mtDNA variants (although we will also consider *de novo* mutation using simple statistical genetics models). The variance of the distribution of heteroplasmies gives the fraction of cells above a given pathological threshold (Figure 1B). Therefore, heteroplasmy variance is related to the number of dysfunctional cells above a phenotypic threshold within a tissue, and both heteroplasmy mean and variance are directly related to tissue physiology. Increases in heteroplasmy variance also increase the number of cells below a given threshold heteroplasmy, which can be advantageous in, *e.g.*, selecting low-heteroplasmy embryos in preimplantation genetic diagnosis for treating mitochondrial disease (Burgstaller *et al.* 2014b; Johnston *et al.* 2015).

**Figure 1:**
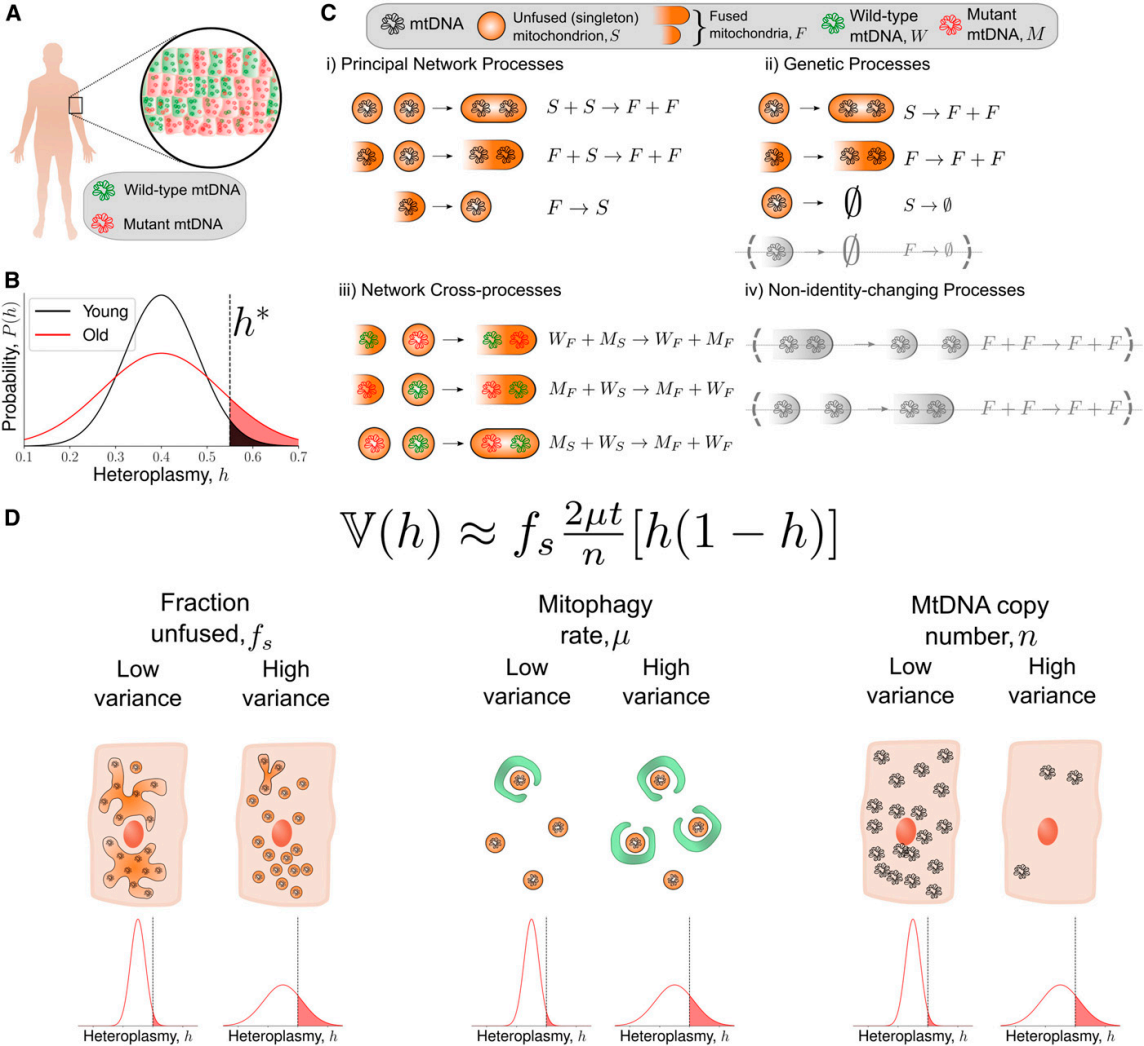
A simple model bridging mitochondrial networks and genetics yields a wide-reaching, analytically obtained description of heteroplasmy variance dynamics. (A) A population of cells from a tissue exhibit intercellular heterogeneity in mitochondrial content: both mutant load (heteroplasmy) and copy number. (B) Intercellular heterogeneity implies that heteroplasmy is described by a probability distribution. Cells above a threshold heteroplasmy ($h^{\text{*}}$, black dashed line) are thought to exhibit a pathological phenotype. The low-variance distribution (black line) has fewer cells above a pathological threshold heteroplasmy than the high-variance distribution (red line). Heteroplasmy is depicted as an approximately normal distribution, as this is the regime in which our approximations below hold: *i.e.*, when the probability of fixation is small. (C) The chemical reaction network we use to model the dynamics of mitochondrial DNA (see main text for a detailed description). mtDNAs are assigned a genetic state of mutant (*M*) or wild type (*W*), and a network state of singleton (*i.e.*, unfused, *S*) or fused (*F*). (D) The central result of our work is, assuming that a cell at time t=0 is at its (deterministic) steady state, heteroplasmy variance (V(h)) approximately increases with time (*t*), mitophagy rate (*μ*), and the fraction of mitochondria that are unfused $\left(f_{s}\right)$, and decreases with mtDNA copy number (*n*). Importantly, V(h) does not depend on the absolute magnitude of the fission–fusion rates. Also see Table S1 for a summary of our key findings.

Mitochondria exist within a network which dynamically fuses and fragments. Although the function of mitochondrial networks remains an open question (Hoitzing *et al.* 2015), it is often thought that a combination of network dynamics and mitochondrial autophagy (termed mitophagy) act in concert to perform quality control on the mitochondrial population (Twig *et al.* 2008; Aryaman *et al.* 2019; Johnston 2019). Observations of pervasive intramitochondrial mtDNA mutation (Morris *et al.* 2017) and universal heteroplasmy in humans (Payne *et al.* 2012) suggest that the power of this quality control may be limited. It has also been suggested that certain mtDNA mutations, such as deletions (Kowald and Kirkwood 2013, 2014, 2018) and some point mutations (Samuels *et al.* 2013; Ye *et al.* 2014; Li *et al.* 2015; Lieber *et al.* 2019), are under the influence of selective effects. However, genetic models without selection have proven valuable in explaining the heteroplasmy dynamics both of functional mutations (Elson *et al.* 2001; Taylor *et al.* 2003; Wonnapinij *et al.* 2008) and polymorphisms without dramatic functional consequences (Birky *et al.* 1983; Ye *et al.* 2014), and in common cases where mean heteroplasmy shifts are small compared to changes in variances [for instance, in germline development (Johnston *et al.* 2015) and postmitotic tissues (Burgstaller *et al.* 2014a)]. Mean changes seem more likely in high-turnover tissues and when mtDNA variants are genetically distant (Burgstaller *et al.* 2014a; Pan *et al.* 2019), suggesting that neutral genetic theory may be useful in understanding the dynamics of the set of functionally mild mutations which accumulate during aging. Furthermore, there currently exists limited evidence for pronounced, universal, selective differences of mitochondrial variants in vivo (Stewart and Larsson 2014; Hoitzing 2017). Neutral genetic theory also provides a valuable null model for understanding mitochondrial genetic dynamics (Chinnery and Samuels 1999; Poovathingal *et al.* 2009; Johnston and Jones 2016), potentially allowing us to better understand and quantify when selection is present. There is thus a set of open questions about how the physical dynamics of mitochondria affect the genetic populations of mtDNA within and between cells under neutral dynamics.

A number of studies have attempted to understand the impact of the mitochondrial network on mitochondrial dysfunction through computer simulation (reviewed in Kowald and Klipp 2014). These studies have suggested the following: that clearance of damaged mtDNA can be assisted by high and functionally selective mitochondrial fusion, or by intermediate fusion and selective mitophagy (Mouli *et al.* 2009); that physical transport of mitochondria can indirectly modulate mitochondrial health through mitochondrial dynamics (Patel *et al.* 2013); that fission–fusion dynamic rates modulate a trade-off between mutant proliferation and removal (Tam *et al.* 2013, 2015); and that if fission is damaging, decelerating fission–fusion cycles may improve mitochondrial quality (Figge *et al.* 2012).

Despite providing valuable insights, these previous attempts to link mitochondrial genetics and network dynamics—while important for breaking ground—have centered around complex computer simulations, making it difficult to deduce general laws and principles. Here, we address this lack of a general theoretical framework linking mitochondrial dynamics and genetics. We take a simpler approach in terms of our model structure (Figure 1C), allowing us to derive explicit, interpretable, mathematical formulas which provide intuitive understanding, and give a direct account for the phenomena which are observed in our model (Figure 1D). This simplified approach ensures that our results hold for a range of variant model structures. Simplified approaches using stochastic modeling have shown success in understanding mitochondrial physiology from a purely genetic perspective (Chinnery and Samuels 1999; Capps *et al.* 2003; Johnston and Jones 2016). Our basic approach also differs from previous modeling attempts, since our model is neutral with respect to genetics (no replicative advantage or selective mitophagy) and the mitochondrial network (no selective fusion). Evidence for negative selection of particular mtDNA mutations has been observed *in vivo* (Ye *et al.* 2014; Morris *et al.* 2017); we therefore extend our analysis to explore selectivity in the context of mitochondrial quality control using our simplified framework.

Here, we reveal the first general mathematical principle linking (physical) network state and (genetic) heteroplasmy statistics (Figure 1D). Our models potentially allow rich interactions between mitochondrial genetic and network dynamics, yet we find that a simple link emerges. For a broad range of situations, the expansion of mtDNA mutants is strongly modulated by network state, such that the rate of increase of heteroplasmy variance, and the rate of accumulation of *de novo* mutation, is proportional to the fraction of unfused mitochondria. We discover that this result stems from the general notion that fusion shields mtDNAs from turnover, since autophagy of large fragments of the mitochondrial network are unlikely, and consequently rescales time. Importantly, we used our model for network dynamics to show that heteroplasmy variance is independent of the absolute magnitude of the fusion and fission rates due to a separation of timescales between genetic and network processes (in contrast to Tam *et al.* 2015). Surprisingly, we find the dependence of heteroplasmy statistics upon network state arises when the mitochondrial population size is controlled through replication, and vanishes when it is controlled through mitophagy, shedding new light on the physiological importance of the mode of mtDNA control. We show that when fusion is selective, intermediate fusion:fission ratios are optimal for the clearance of mutated mtDNAs (in contrast to Mouli *et al.* 2009). When mitophagy is selective, complete fragmentation of the network results in the most effective elimination of mitochondrial mutants (in contrast to Mouli *et al.* 2009). We also confirm that mitophagy and mitochondrial DNA copy number affect the rate of accumulation of *de novo* mutations (Johnston and Jones 2016), see Supplemental Material, Table S1 for a summary of our key findings. We suggest that pharmacological interventions which promote fusion, slow mitophagy, and increase copy number earlier in development may slow the rate of accumulation of pathologically mutated cells, with implications for mitochondrial disease and aging.

## Materials and Methods

### Stochastic modeling of the coupling between genetic and network dynamics of mtDNA populations

Our modeling approach takes a chemical master equation perspective by combining a general model of neutral genetic drift (for instance, see Chinnery and Samuels 1999 and Johnston and Jones 2016) with a model of mitochondrial network dynamics. We seek to understand the influence of the mitochondrial network upon mitochondrial genetics. The network state itself is influenced by several factors including metabolic poise and the respiratory state of mitochondria (Szabadkai *et al.* 2006; Hoitzing *et al.* 2015; Mishra and Chan 2016), which we do not consider explicitly here. We consider the existence of two mitochondrial alleles, wild-type (*W*) and mutant (*M*), existing within a postmitotic cell without cell division, with mtDNAs undergoing turnover [or “relaxed replication” (Stewart and Chinnery 2015)]. mtDNAs exist within mitochondria, which undergo fusion and fission. We therefore assign mtDNAs a network state: fused (*F*) or unfused (we term “singleton,” *S*). This representation of the mitochondrial network allows us to include the effects of the mitochondrial network in a simple way, without the need to resort to a spatial model or consider the precise network structure, allowing us to make analytic progress and derive interpretable formulas in a more general range of situations.

Our model can be decomposed into three notional blocks (Figure 1C). First, the principal network processes denote fusion and fission of mitochondria containing mtDNAs of the same allele

$$X_{S}+X_{S}\overset{\gamma }{\to }X_{F}+X_{F}$$ (1)

$$X_{F}+X_{S}\overset{\gamma }{\to }X_{F}+X_{F}$$ (2)

$$X_{F}\overset{\beta }{\to }X_{S},$$ (3)

where *X* denotes either a wild-type (*W*) or a mutant (*M*) mtDNA (therefore a set of chemical reactions analogous to Equations 1–3 exist for both DNA species). *γ* and *β* are the stochastic rate constants for fusion and fission respectively.

Second, mtDNAs are replicated and degraded through a set of reactions termed genetic processes. A central assumption is that all degradation of mtDNAs occur through mitophagy, and that only small pieces of the mitochondrial network are susceptible to mitophagy; for parsimony we take the limit of only the singletons being susceptible to mitophagy:

$$X_{S}\overset{\lambda }{\to }X_{F}+X_{F}$$ (4)

$$X_{F}\overset{\lambda }{\to }X_{F}+X_{F}$$ (5)

$$X_{S}\overset{\mu }{\to }\mathrm{\emptyset ,}$$ (6)

where λ and *μ* are the replication and mitophagy rates, respectively, which are shared by both *W* and *M* resulting in a so-called “neutral” genetic model. Equation 6 denotes removal of the species from the system. The effect of allowing nonzero degradation of fused species is discussed in the Supplemental Material (see Equation S68 and Figure S3E). Replication of a singleton changes the network state of the mtDNA into a fused species, since replication occurs within the same membrane-bound organelle. An alternative model of singletons which replicate into singletons, thereby associating mitochondrial replication with fission (Lewis *et al.* 2016), leaves our central result (Figure 1D) unchanged (see Equation S67). The system may be considered neutral since both *W* and *M* possess the same replication and degradation rates per molecule of mtDNA at any instance in time.

Finally, mtDNAs of different genotypes may interact through fusion via a set of reactions we term network cross-processes:

$$W_{F}+M_{S}\overset{\gamma }{\to }W_{F}+M_{F}$$ (7)

$$M_{F}+W_{S}\overset{\gamma }{\to }M_{F}+W_{F}$$ (8)

$$W_{S}+M_{S}\overset{\gamma }{\to }W_{F}+M_{F}.$$ (9)

Any fusion or fission event which does not involve the generation or removal of a singleton leaves our system unchanged; we term such events as nonidentity-changing processes, which can be ignored in our system (see *Rate renormalization* in the Supplemental Material for a discussion of rate renormalization). We have neglected *de novo* mutation in the model description above (although we will consider *de novo* mutation using a modified infinite sites Moran model below).

We found that treating λ as a constant led to instability in total copy number (see *Constant rates yield unstable copy numbers for a model describing mtDNA genetic and network dynamics* in the Supplemental Material), which is not credible. We therefore favored a state-dependent replication rate such that copy number is controlled to a particular value, as has been done by previous authors (Chinnery and Samuels 1999; Capps *et al.* 2003; Johnston and Jones 2016). Allowing lower-case variables to denote the copy number of their respective molecular species, we will focus on a linear replication rate of the form (Hoitzing 2017; Hoitzing *et al.* 2019):

$$\lambda =\lambda \left(w_{T},m_{T}\right)=\mu +b\left(\kappa -\left(w_{T}+\delta m_{T}\right)\right),$$ (10)

where $w_{T}=w_{s}+w_{f}$ is the total wild-type copy number, and similarly for $m_{T}$. The lower-case variables $w_{s}$, $w_{f}$, $m_{s}$, and $m_{f}$ denote the copy numbers of the corresponding chemical species ($W_{S}$, $W_{F}$, $M_{S}$, and $M_{F}$). *b* is a parameter which determines the strength with which total copy number is controlled to a target copy number, and *κ* is a parameter which is indicative of (but not equivalent to) the steady-state copy number. *δ* indicates the relative contribution of mutant mtDNAs to the control strength and is linked to the “maintenance of wild-type” hypothesis (Durham *et al.* 2007; Stewart and Chinnery 2015). When 0≤δ<1, and both mutant and wild-type species are present, mutants have a lower contribution to the birth rate than wild types. When wild types are absent, the population size will be larger than when there are no mutants: hence mutants have a higher carrying capacity in this regime. We have modeled the mitophagy rate as constant per mtDNA. We do, however, explore relaxing this constraint below by allowing mitophagy to be a function of state, and also affect mutants differentially under quality control. *λ* may be rewritten as $\lambda =k_{1}+k_{2}w_{T}+k_{3}m_{T}$ for constants $k_{i}$, and so only consists of three independent parameters. However we will retain *λ* in the form of Equation 10 since the parameters *μ*, *b*, *κ*, and *δ* have the distinct physiological meanings described above (Hoitzing 2017; Hoitzing *et al.* 2019). Furthermore, *λ* may in general also depend on other cellular features such as mitochondrial reactive oxygen species. Here, we seek to explain mitochondrial behavior under a simple set of governing principles, but our approach can naturally be combined with a description of these additional factors to build a more comprehensive model. Analogs of this model (without a network) have been applied to mitochondrial systems (Chinnery and Samuels 1999; Capps *et al.* 2003). Overall, our simple model consists of 4 species $\left(W_{S},W_{F},M_{S},M_{F}\right)$, 6 independent parameters, and 15 reactions, and captures the central property that mitochondria fragment before degradation (Twig *et al.* 2008).

Throughout this work, we define heteroplasmy as the mutant-allele fraction per cell of a mitochondrially encoded variant (Wonnapinij *et al.* 2008; Samuels *et al.* 2010; Aryaman *et al.* 2019):

$$h\left(x\right)=\left(m_{s}+m_{f}\right)/\left(w_{s}+w_{f}+m_{s}+m_{f}\right),$$ (11)

where $x=\left(w_{s},w_{f},m_{s},m_{f}\right)$ is the state of the system (not to be confused with mitochondrial “respiratory states”). Hence, a heteroplasmy of h=1 denotes a cell with 100% mutant mtDNA (*i.e.*, a homoplasmic cell in the mutant allele). Arguably, mutant-allele fraction would be a more precise description of Equation 11 but we retain the use of heteroplasmy for consistency. To convert to a definition of heteroplasmy which is maximal when the mutant allele fraction is 50%, one may simply use the conversion 0.5−|h(x)−0.5|.

### Statistical analysis

In Figure S3B and Figure S4, A–I, we compare Equation 13 and Equation S72 to stochastic simulations for various parameterizations and replication/degradation rates. To quantify the accuracy of these equations in predicting V(h,t), we define the following error metric ϵ

$$\epsilon =\left|1-\frac{\dot{V}{(h,t)}_{\text{Th}}}{E_{t}\left(\dot{V}{\left(h,t\right)}_{\text{Sim}}\right)}\right|,$$ (12)

where $\dot{V}\left(h,t\right)$ is the time derivative of heteroplasmy variance with subscripts denoting theory (Th) and simulation (Sim). An expectation over time $\left(E_{t}\right)$ is taken for the stochastic simulations, whereas $\dot{V}\left(h,t\right)$ is a scalar quantity for Equation 13 and Equation S72.

### Data availability

Code for simulations and analysis can be accessed at https://GitHub.com/ImperialCollegeLondon/MitoNetworksGenetics. Supplemental material available at FigShare: https://doi.org/10.25386/genetics.8343830.

## Results

### Mitochondrial network state rescales the linear increase of heteroplasmy variance over time, independently of fission–fusion rate magnitudes

**Figure 2:**
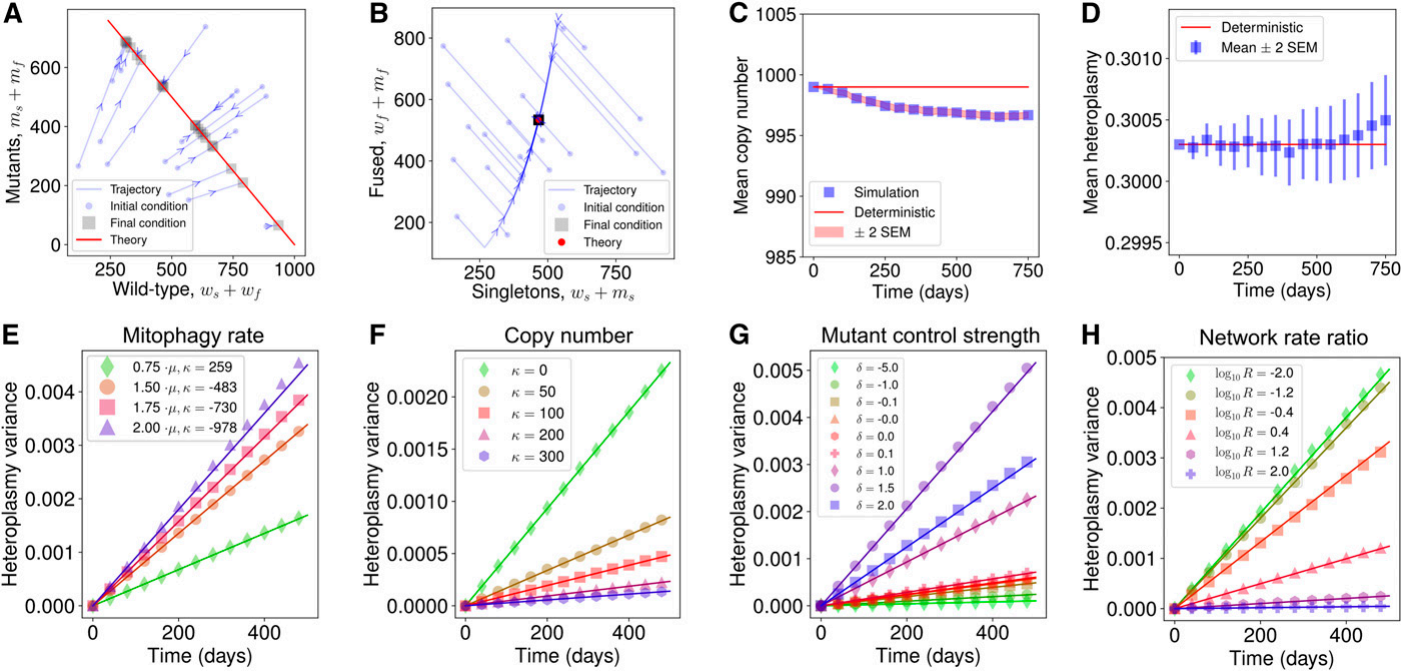
General mathematical principles linking heteroplasmy variance to network dynamics. (A) Wild-type and mutant copy numbers and (B) fused and unfused copy numbers both move toward a line of steady states under a deterministic model, as indicated by arrows. In stochastic simulation, (C) mean copy number is initially slightly perturbed from the deterministic treatment of the system and then remains constant, while (D) mean heteroplasmy remains invariant with time (see Equation S61). (E–H) We show that Equation 13 holds across many cellular circumstances: lines give analytic results, points are from stochastic simulation. Heteroplasmy variance behavior is successfully predicted for varying (E) mitophagy rate, (F) steady-state copy number, (G) mutation sensing, and (H) fusion rate. In H, fusion and fission rates are redefined as $\gamma \to \gamma_{0}MR$ and $\beta \to \beta_{0}M,$ where *M* and *R* denote the relative magnitude and ratio of the network rates, and $\gamma_{0},\beta_{0}$ denote the nominal parameterizations of the fusion and fission rates, respectively (see Table S2). Figure S3D shows a sweep of *M* over the same logarithmic range when R=1. See Figure S4, A–I, and Table S3 for parameter sweeps numerically demonstrating the generality of the result for different mtDNA control modes.

We first performed a deterministic analysis of the system presented in Equations 1–10 by converting the reactions into an analogous set of four coupled ordinary differential equations (see Equations S29–S32) and choosing a biologically motivated approximate parameterization (which we will term the “nominal” parameterization, see *Choice of nominal parametrization* in the Supplemental Material and Table S2). Figure 2, A and B, show that copy numbers of each individual species change in time such that the state approaches a line of steady states (Equations S34–S36), as seen in other neutral genetic models (Capps *et al.* 2003; Hoitzing 2017). Upon reaching this line, total copy number remains constant (Figure S2A) and the state of the system ceases to change with time. This is a consequence of performing a deterministic analysis, which neglects stochastic effects, and our choice of replication rate in Equation 10 which decreases with total copy number when $w_{T}+\delta m_{T}>\kappa ,$ and vice versa, guiding the total population to a fixed total copy number. Varying the fission (*β*) and fusion (*γ*) rates revealed a negative linear relationship between the steady-state fraction of singletons and copy number (Figure S2B).

We may also simulate the system in Equations 1–9 stochastically, using the stochastic simulation algorithm (Gillespie 1976), which showed that mean copy number is slightly perturbed from the deterministic prediction due to the influence of variance upon the mean (Grima *et al.* 2011; Hoitzing 2017) (Figure 2C). The stationarity of total copy number is a consequence of using δ=1 for our nominal parameterization (*i.e.*, the line of steady states is also a line of constant copy number). Choosing δ≠1 results in a difference in carrying capacities between the two species and nonstationarity of mean total copy number, as trajectories spread along the line of steady states to different total copy numbers. Copy number variance initially increases since trajectories are all initialized at the same state, but plateaus because trajectories are constrained in their copy number to remain near the attracting line of steady states (Figure S3A). Mean heteroplasmy remains constant through time under this model (Figure 2D; see Birky *et al.* 1983). This is unsurprising since each species possesses the same replication and degradation rate, so neither species is preferred.

From stochastic simulations we observed that, for sufficiently short times, heteroplasmy variance increases approximately linearly through time for a range of parameterizations (Figure 2, E–H), which is in agreement with recent single-cell oocyte measurements in mice (Burgstaller *et al.* 2018). Previous work has also shown a linear increase in heteroplasmy variance through time for purely genetic models of mtDNA dynamics (see Johnston and Jones 2016). We sought to understand the influence of mitochondrial network dynamics upon the rate of increase of heteroplasmy variance.

To this end, we analytically explored the influence of mitochondrial dynamics on mtDNA variability. Assuming that the state of the system above is initialized at its deterministic steady state $\left(x\left(t=0\right)=x_{\text{ss}}\right)$, we took the limit of large mtDNA copy numbers (mtCNs), fast fission–fusion dynamics, and applied a second-order truncation of the Kramers–Moyal expansion (Gardiner 1985) to the chemical master equation describing the dynamics of the system (see Supplemental Material). This yielded a stochastic differential equation for heteroplasmy, via Itô’s formula (Jacobs 2010). Upon forcing the state variables onto the steady-state line (Constable *et al.* 2016), we derived Equation S63, which may be approximated for sufficiently short times as

$$V\left(h\right)\approx f_{s}\left(x\right)\frac{2\mu t}{n\left(x\right)}h\left(x\right)\left(1-h\left(x\right)\right)|{}_{x=x_{\text{ss}}}.$$ (13)

Here, V(h) is the variance of heteroplasmy, *μ* is the mitophagy rate, n(x) is the total copy number, and $f_{s}\left(x\right)$ is the fraction of unfused (singleton) mtDNAs, and is thus a measure of the fragmentation of the mitochondrial network. $x_{\text{ss}}$ is the (deterministic) steady state of the system. Equation 13 demonstrates that mtDNA heteroplasmy variance increases approximately linearly with time (*t*) at a rate scaled by the fraction of unfused mitochondria, mitophagy rate, and inverse population size. We find that Equation 13 closely matches heteroplasmy variance dynamics from stochastic simulation, for sufficiently short times after initialization, for a variety of parameterizations of the system (Figure 2, E–H, and Figure S5).

To our knowledge, Equation 13 reflects the first analytical principle linking mitochondrial dynamics and the cellular population genetics of mtDNA variance. Its simple form allows several intuitive interpretations. As time progresses, replication and degradation of both species occurs, allowing the ratio of species to fluctuate; hence we expect V(h) to increase with time according to random genetic drift (Figure 2, E–H). The rate of occurrence of replication/degradation events is set by the mitophagy rate *μ*, since degradation events are balanced by replication rates to maintain population size; hence, random genetic drift occurs more quickly if there is a larger turnover in the population (Figure 2E). We expect V(h) to increase more slowly in large population sizes, since the birth of, *e.g.*, one mutant in a large population induces a small change in heteroplasmy (Figure 2F). The factor of h(1−h) encodes the state dependence of heteroplasmy variance, exemplified by the observation that if a cell is initialized at h=0 or h=1, heteroplasmy must remain at its initial value (since the model above does not consider *de novo* mutation, see below) and so heteroplasmy variance is zero. Furthermore, the rate of increase of heteroplasmy variance is maximal when a cell’s initial value of heteroplasmy is 0.5. In Figure 2G, we show that Equation 13 is able to recapitulate the rate of heteroplasmy variance increase across different values of *δ*, which are hypothesized to correspond to different replicative sensing strengths of different mitochondrial mutations (Hoitzing 2017). We also show in Figure S3, B and C, that Equation 13 is robust to the choice of feedback control strength *b* in Equation 10. n(x), f(x), and h(x) in Equation 13 are not independent degrees of freedom in this model: they are functions of the state vector **x**, where **x** is determined by the parameterization and initial conditions of the model. Hence, the parameter sweeps in Figure 2, E–H, and Figure S3, B and C, also implicitly vary over these functions of state by varying the steady state $x_{\text{ss}}$.

In Equation 6, we have made the important assumption that only unfused mitochondria can be degraded via mitophagy, as seen by Twig *et al.* (2008), hence the total propensity of mtDNA turnover is limited by the number of mtDNAs which are actually susceptible to mitophagy. Strikingly, we find that the dynamics of heteroplasmy variance are independent of the absolute rate of fusion and fission, only depending on the fraction of unfused mtDNAs at any particular point in time (see Figure 2H and Figure S3D). This observation, which contrasts with the model of Tam *et al.* (2013, 2015) (see *Discussion*), arises from the observation that mitochondrial network dynamics are much faster than replication and degradation of mtDNA, by around a factor of $\beta /\mu \approx 10^{3}$ (see Table S2), resulting in the existence of a separation of timescales between network and genetic processes. In the derivation of Equation 13, we have assumed that fission–fusion rates are infinite, which simplifies V(h) into a form which is independent of the magnitude of the fission–fusion rate. A parameter sweep of the magnitude and ratio of the fission–fusion rates reveals that, if the fusion and fission rates are sufficiently small, Equation 13 breaks down and V(h) gains dependence upon the magnitude of these rates (see Figure S4A). This regime only appears, however, for network rates which are ∼100-times smaller than the biologically motivated nominal parameterization shown in Figure 2, A–D, where the fission–fusion rate becomes comparable to the mitophagy rate. Since fission–fusion takes place on a faster timescale than mtDNA turnover, we may neglect this region of parameter space as being implausible.

Equation 13 can be viewed as describing the “quasi-stationary state” where the probability of extinction of either allele is negligible (Johnston and Jones 2016). On longer timescales, or if mtDNA half-life is short (Poovathingal *et al.* 2012), the probability of fixation becomes appreciable. In this case, Equation 13 overestimates V(h) as heteroplasmy variance gradually becomes sublinear with time (see Figure S5, C and D). This is evident through inspection of Equation S63, which shows that cellular trajectories which reach h=0 or h=1 cease to diffuse in heteroplasmy space, and so heteroplasmy variance cannot increase indefinitely. Consequently, the depiction of heteroplasmy variance in Figure 1, B and D, as being approximately normally distributed corresponds to the regime in which our approximation holds, and is a valid subset of the behaviors displayed by heteroplasmy dynamics under more sophisticated models [*e.g.*, the Kimura distribution (Kimura 1955; Wonnapinij *et al.* 2008)]. Further analytical developments may be possible to take into account extinction (*e.g.*, see Wonnapinij *et al.* 2008 and Assaf and Meerson 2010). However, the linear regime for heteroplasmy variance has been observed to be a substantial component of mtDNA dynamics in, *e.g.*, mouse oocytes (Burgstaller *et al.* 2018).

### The influence of mitochondrial dynamics upon heteroplasmy variance under different models of genetic mtDNA control

To demonstrate the generality of this result, we explored several alternative forms of cellular mtDNA control (Johnston and Jones 2016). We found that when copy number is controlled through the replication rate function [*i.e.*, λ=λ(x), μ= constant], when the fusion and fission rates were high and the fixation probability [P(h=0) or P(h=1)] was negligible, Equation 13 accurately described V(h) across all of the replication rates investigated (see Figure S4, A–F). The same mathematical argument to show Equation 13 for the replication rate in Equation 10 may be applied to these alternative replication rates where a closed-form solution for the deterministic steady state may be written down (see *Deriving an ODE description of the mitochondrial network system* in the Supplemental Material). Interestingly, when copy number is controlled through the degradation rate [*i.e.*, λ=constant,μ=μ(x)], heteroplasmy variance loses its dependence upon network state entirely and the $f_{s}$ term is lost from Equation 13 (see Equation S72 and Figure S4, G–I). A similar mathematical argument was applied to reveal how this dependence is lost (see *Proof of heteroplasmy relation for linear feedback control* in the Supplemental Material).

To provide an intuitive account for why control in the replication rate *vs.* control in the degradation rate determines whether or not heteroplasmy variance has network dependence, we investigated a time-rescaled form of the Moran process (see *A modified Moran process may account for the alternative forms of heteroplasmy variance dynamics under different models of genetic mtDNA control* in the Supplemental Material). The Moran process is structurally much simpler than the model presented above, to the point of being unrealistic, in that the mitochondrial population size is constrained to be constant between consecutive time steps. Despite this, the modified Moran process proved to be insightful. We find that, when copy number is controlled through the replication rate, the absence of death in the fused subpopulation means the timescale of the system (being the time to the next death event) is proportional to $f_{s}$. In contrast, when copy number is controlled through the degradation rate, the presence of a constant birth rate in the entire population means the timescale of the system (being the time to the next birth event) is independent of $f_{s}$ (see Equation S84 and surrounding discussion).

### Control strategies against mutant expansions

In this study, we have argued that the rate of increase of heteroplasmy variance, and therefore the rate of accumulation of pathologically mutated cells within a tissue, increases with mitophagy rate (*μ*), decreases with total mtCN per cell (*n*), and increases with the fraction of unfused mitochondria (termed singletons, $f_{s}$), see Equation 13. Below, we explore how biological modulation of these variables influences the accumulation of mutations. We use this new insight to propose three classes of strategy to control mutation accumulation and hence address associated issues in aging and disease, and discuss these strategies through the lens of existing biological literature.

#### Targeting network state against mutant expansions:

**Figure 3:**
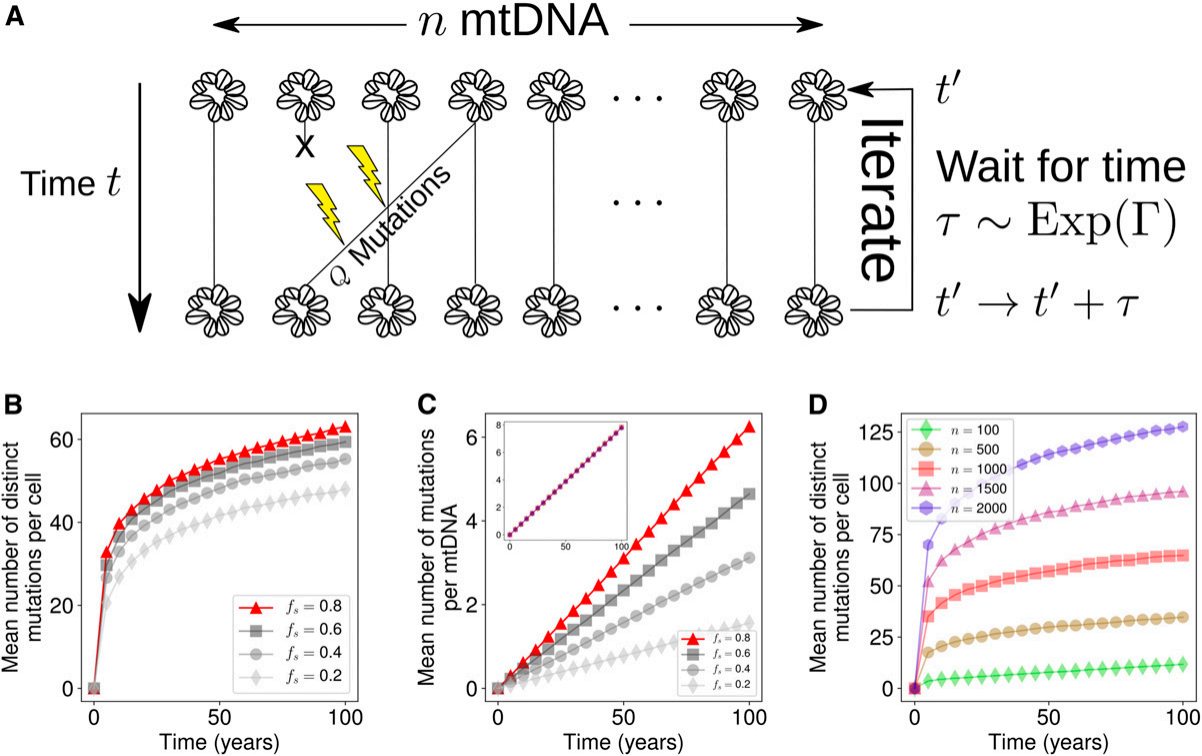
Rate of *de novo* mutation accumulation is sensitive to the network state/mitophagy rate and copy number for a time-rescaled infinite sites Moran model. (A) An infinite sites Moran model where *Q* mutations occur per Moran step (see Equation 14). (B–D) Influence of our proposed intervention strategies. (B) Mean number of distinct mutations increases with the fraction of unfused mitochondria. This corresponds to a simple rescaling of time, so all but one of the parameterizations are shown in gray. (C) The mean number of mutations per mtDNA also increases with the fraction of unfused mitochondria. Inset shows that the mean number of mutations per mtDNA is independent of the number of mtDNAs per cell; values of *n* are the same as in D. (D) Mean number of mutations per cell increases according to the population size of mtDNAs. Standard error in the mean is too small to visualize, so error bars are neglected, given $10^{3}$ realizations.

To explore the role of the mitochondrial network in the accumulation of *de novo* mutations, we invoked an infinite sites Moran model (Kimura 1969) (see Figure 3A). Single cells were modeled over time as having a fixed mitochondrial copy number (*n*), and at each time step one mtDNA is randomly chosen for duplication and one (which can be the same) for removal. The individual replicated incurs *Q de novo* mutations, where *Q* is binomially distributed according to

$$Q∼\text{Binomial}\left(L_{\text{mtDNA}},\eta \right),$$ (14)

where Binomial(N,p) is a binomial random variable with *N* trials and probability *p* of success. $L_{\text{mtDNA}}=16569$ is the length of mtDNA in base pairs and $\eta =5.6\times 10^{-7}$ is the mutation rate per base pair per doubling (Zheng *et al.* 2006); hence each base pair is idealized to have an equal probability of mutation upon replication. In Equation S83 we argue that when population size is controlled in the replication rate, the interevent rate (Γ) of the Moran process is effectively rescaled by the fraction of unfused mitochondria, *i.e.*, $\text{Γ}=\mu nf_{s}$, which we apply here.

Figure 3B shows that in the infinite sites model, the consequence of Equation S83 is that the rate of accumulation of mutations per cell reduces as the mitochondrial network becomes more fused, as does the mean number of mutations per mtDNA (Figure 3C). These observations are intuitive: since fusion serves to shield the population from mitophagy, mtDNA turnover slows down, and therefore there are fewer opportunities for replication errors to occur per unit time. Different values of $f_{s}$ in Figure 3, B and C, therefore correspond to a rescaling of time, *i.e.*, stretching of the time axis. The absolute number of mutations predicted in Figure 3B may overestimate the true number of mutations per cell (and of course depends on our choice of mutation rate), since a subset of mutations will experience either positive or negative selection. However, quantification of the number of distinct mitochondrial mutants in single cells remains underexplored, as most mutations will have a variant allele fraction close to 0 or 100% (Birky *et al.* 1983), which are challenging to measure, especially through bulk sequencing.

A study by Chen *et al.* (2010) observed the effect of deletion of two proteins which are involved in mitochondrial fusion (Mfn1 and Mfn2) in mouse skeletal muscle. Although knock-out studies present difficulties in extending their insights into the physiological case, the authors observed that fragmentation of the mitochondrial network induced severe depletion of mtCN (which we also observed in Figure S2B). Furthermore, the authors observed that the number of mutations per base pair increased upon fragmentation, which we also observed in the infinite sites model where fragmentation effectively results in a faster turnover of mtDNA (Figure 3C).

Our models predict that promoting mitochondrial fusion has a twofold effect: first, it slows the increase of heteroplasmy variance (see Equation 13 and Figure 2H); second, it reduces the rate of accumulation of distinct mutations (see Figure 3, B and C). These two effects are both a consequence of mitochondrial fusion rescaling the time to the next turnover event, and therefore the rate of random genetic drift. As a consequence, this simple model suggests that promoting fusion earlier in development (assuming mean heteroplasmy is low) could slow down the accumulation and spread of mitochondrial mutations, and perhaps slow aging.

If we assume that fusion is selective in favor of wild-type mtDNAs, which appears to be the case at least for some mutations under therapeutic conditions (Suen *et al.* 2010; Kandul *et al.* 2016), we predict that a balance between fusion and fission is the most effective means of removing mutant mtDNAs (see below), perhaps explaining why mitochondrial networks are often observed to exist as balanced between mitochondrial fusion and fission (Sukhorukov *et al.* 2012; Zamponi *et al.* 2018). In contrast, if selective mitophagy pathways are induced then promoting fragmentation is predicted to accelerate the clearance of mutants (see below).

#### Targeting mitophagy rate against mutant expansions:

Alterations in the mitophagy rate *μ* have a comparable effect to changes in $f_{s}$ in terms of reducing the rate of heteroplasmy variance (see Equation 13) and the rate of *de novo* mutation (Figure 3, B and C) since they both serve to rescale time. Our theory therefore suggests that inhibition of basal mitophagy may be able to slow down the rate of random genetic drift, and perhaps healthy aging, by locking in low levels of heteroplasmy. Indeed, it has been shown that mouse oocytes (Boudoures *et al.* 2017) as well as mouse hematopoietic stem cells (de Almeida *et al.* 2017) have comparatively low levels of mitophagy, which is consistent with the idea that these pluripotent cells attempt to minimize genetic drift by slowing down mtDNA turnover. A previous modeling study has also shown that mutation frequency increases with mitochondrial turnover (Poovathingal *et al.* 2009).

Alternatively, it has also been shown that the presence of heteroplasmy, in genotypes which are healthy when present at 100%, can induce fitness disadvantages (Acton *et al.* 2007; Sharpley *et al.* 2012; Bagwan *et al.* 2018). In cases where heteroplasmy itself is disadvantageous, especially in later life where such mutations may have already accumulated, accelerating heteroplasmy variance increase to achieve fixation of a species could be advantageous. However, this will not avoid cell-to-cell variability, and the physiological consequences for tissues of such mosaicism is unclear.

#### Targeting copy number against mutant expansions:

To investigate the role of mtCN on the accumulation of *de novo* mutations, we set $f_{s}=1$ such that Γ=μn (*i.e.*, a standard Moran process). We found that varying mtCN did not affect the mean number of mutations per molecule of mtDNA (Figure 3C, inset). However, as the population size becomes larger, the total number of distinct mutations increases accordingly (Figure 3D). In contrast to our predictions, a recent study by Wachsmuth *et al.* (2016) found a negative correlation between mtCN and the number of distinct mutations in skeletal muscle. However, Wachsmuth *et al.* (2016) also found a correlation between the number of distinct mutations and age, in agreement with our model. Furthermore, the authors used partial regression to find that age was more explanatory than mtCN in explaining the number of distinct mutations, suggesting age as a confounding variable to the influence of copy number. Our work shows that, in addition to age and mtCN, turnover rate and network state also influence the proliferation of mtDNA mutations. Therefore, one would ideally account for these four variables jointly, to fully constrain our model.

A study of single neurons in the substantia nigra of healthy human individuals found that mtCN increased with age (Dölle *et al.* 2016). Furthermore, mice engineered to accumulate mtDNA deletions through faulty mtDNA replication (Trifunovic *et al.* 2004) display compensatory increases in mtCN (Perier *et al.* 2013), which potentially explains the ability of these animals to resist neurodegeneration. It is possible that the observed increase in mtCN in these two studies is an adaptive response to slow down random genetic drift (see Equation 13). In contrast, mtCN reduces with age in skeletal muscle (Wachsmuth *et al.* 2016), as well as in a number of other tissues such as pancreatic islets (Cree *et al.* 2008) and peripheral blood cells (Mengel-From *et al.* 2014). Given the beneficial effects of increased mtCN in neurons, long-term increases in mtCN could delay other age-related pathological phenotypes.

### Optimal mitochondrial network configurations for mitochondrial quality control

While the above models of mtDNA dynamics are neutral (*i.e.*, *m* and *w* share the same replication and degradation rates), it is often proposed that damaged mitochondria may experience a higher rate of degradation (Kim *et al.* 2007; Narendra *et al.* 2008). There are two principal ways in which selection may occur on mutant species. First, mutant mitochondria may be excluded preferentially from the mitochondrial network in a background of unbiased mitophagy. If this is the case, mutants would be unprotected from mitophagy for longer periods of time than wild types, and therefore be at greater hazard of degradation. We can alter the fusion rate (*γ*) in the mutant analogs of Equations 1 and 2 and Equations 7–9 by writing

$$\gamma \to \gamma /\left(1+\epsilon_{f}\right)$$ (15)

for all fusion reactions involving one or more mutant mitochondria where $\epsilon_{f}>0$. The second potential selective mechanism we consider is selective mitophagy. In this case, the degradation rate of mutant mitochondria is larger than wild types, *i.e.*, we modify the mutant degradation reaction to

$$M_{S}\overset{\mu (1+\epsilon_{m})}{\to }\emptyset$$ (16)

for $\epsilon_{m}>0$.

**Figure 4:**
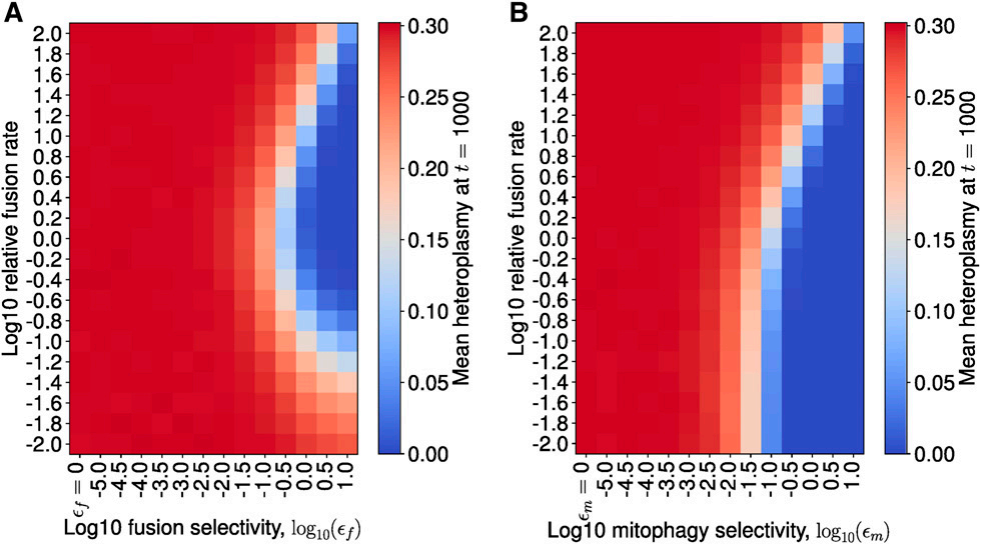
Selective fusion implies intermediate fusion rates are optimal for mutant clearance, whereas selective mitophagy implies complete fission is optimal. Numerical exploration of the shift in mean heteroplasmy for varying fusion:fission ratio, across different selectivity strengths. Stochastic simulations for mean heteroplasmy, evaluated at 1000 days, with an initial condition of h=0.3 and n=1000; the state was initialized on the steady-state line for the case of $\epsilon_{f}=\epsilon_{m}=0$, for $10^{4}$ iterations. (A) For selective fusion (see Equation 15), for each value of fusion selectivity $\left(\epsilon_{f}\right)$, the fusion rate (*γ*) was varied relative to the nominal parameterization (see Table S2). When $\epsilon_{f}>0$, the largest reduction in mean heteroplasmy occurs at intermediate values of the fusion rate; a deterministic treatment reveals this to be true for all fusion selectivities investigated (see Figure S7). (B) For selective mitophagy (see Equation 16), when mitophagy selectivity $\epsilon_{m}>0$, a lower mean heteroplasmy is achieved and the lower the fusion rate (until mean heteroplasmy = 0 is achieved). Hence, complete fission is the optimal strategy for selective mitophagy.

In these two settings, we explore how varying the fusion rate for a given selectivity ($\epsilon_{f}$ and $\epsilon_{m}$) affects the extent of reduction in mean heteroplasmy. Figure 4A shows that, in the context of selective fusion $\left(\epsilon_{f}>0\right)$ and nonselective mitophagy $\left(\epsilon_{m}=0\right),$ the optimal strategy for clearance of mutants is to have an intermediate fusion:fission ratio. This was observed for all fusion selectivities investigated (see Figure S7). Intuitively, if the mitochondrial network is completely fused then, due to mitophagy only acting upon smaller mitochondrial units, mitophagy cannot occur, so mtDNA turnover ceases and heteroplasmy remains at its initial value. In contrast, if the mitochondrial network completely fissions, there is no mitochondrial network to allow the existence of a quality control mechanism: both mutants and wild types possess the same probability per unit time of degradation, so mean heteroplasmy does not change. Since both extremes result in no clearance of mutants, the optimal strategy must be to have an intermediate fusion:fission ratio.

In contrast, in Figure 4B, in the context of nonselective fusion $\left(\epsilon_{f}=0\right)$ and selective mitophagy $\left(\epsilon_{m}>0\right)$, the optimal strategy for clearance of mutants is to completely fission the mitochondrial network. Intuitively, if mitophagy is selective, then the more mtDNAs which exist in fragmented organelles, the greater the number of mtDNAs which are susceptible to selective mitophagy, the greater the total rate of selective mitophagy, and the faster the clearance of mutants.

## Discussion

In this work, we sought to unify our understanding of three aspects of mitochondrial physiology—the mitochondrial network state, mitophagy, and copy number—with genetic dynamics. The principal virtue of our modeling approach is its simplified nature, which makes general, analytic, quantitative insights available for the first time. In using parsimonious models, we are able to make the first analytic link between the mitochondrial network state and heteroplasmy dynamics. This is in contrast to other computational studies in the field, whose structural complexity makes analytic progress difficult and accounting for their predicted phenomena correspondingly more challenging.

Our bottom-up modeling approach allows for potentially complex interactions between the physical (network) and genetic mitochondrial states of the cell, yet a simple connection emerged from our analysis. We found, for a wide class of models of postmitotic cells, that the rate of linear increase of heteroplasmy variance is modulated in proportion to the fraction of unfused mitochondria (see Equation 13). The general notion that mitochondrial fusion shields mtDNAs from turnover, and consequently serves to rescale time, emerges from our analysis. This rescaling of time only holds when mitochondrial copy numbers are controlled through a state-dependent replication rate, and vanishes if copy numbers are controlled through a state-dependent mitophagy rate. We have presented the case of copy-number control in the replication rate as being a more intuitive model than control in the degradation rate. The former has the interpretation of biogenesis being varied to maintain a constant population size, with all mtDNAs possessing a characteristic lifetime. The latter has the interpretation of all mtDNA molecules being replicated with a constant probability per unit time, regardless of how large or small the population size is, and changes in mitophagy acting to regulate population size. Such a control strategy seems wasteful in the case of stochastic fluctuations resulting in a population size which is too large, and potentially slow if fluctuations result in a population size which is too small. Furthermore, control in the replication rate means that the mitochondrial network state may act as an additional axis for the cell to control heteroplasmy variance (Figure 2) and the rate of accumulation of *de novo* mutations (Figure 3, B and C). Single-mtDNA tracking through confocal microscopy in conjunction with mild mtDNA depletion could shed light on whether the probability of degradation per unit time per mtDNA varies when mtCN is perturbed, and therefore provide evidence for or against these two possible control strategies.

Our observations provide a substantial change in our understanding of mitochondrial genetics, as it suggests that the mitochondrial network state, in addition to mitochondrial turnover and copy number, must be accounted for to predict the rate of spread of mitochondrial mutations in a cellular population. Crucially, through building a model that incorporates mitochondrial dynamics, we find that the dynamics of heteroplasmy variance is independent of the absolute rate of fission–fusion events, since network dynamics occur ∼$10^{3}$-times faster than mitochondrial turnover, inducing a separation of timescales. The independence of the absolute rate of network dynamics makes way for the possibility of gaining information about heteroplasmy dynamics via the mitochondrial network, without the need to quantify absolute fission–fusion rates (for instance through confocal micrographs to quantify the fraction of unfused mitochondria). By linking with classical statistical genetics, we find that the mitochondrial network also modulates the rate of accumulation of *de novo* mutations, also due to the fraction of unfused mitochondria serving to rescale time. We find that, in the context of mitochondrial quality control through selective fusion, an intermediate fusion:fission ratio is optimal due to the finite selectivity of fusion. This latter observation perhaps provides an indication for the reason why we observe mitochondrial networks in an intermediate fusion state under physiological conditions (Sukhorukov *et al.* 2012; Zamponi *et al.* 2018).

We have, broadly speaking, considered neutral models of mtDNA genetic dynamics. It is, however, typically suggested that increasing the rate of mitophagy promotes mtDNA quality control and therefore shrinks the distribution of heteroplasmies toward 0% mutant (see Equations 15 and 16). If mitophagy is able to change mean heteroplasmy, then a neutral genetic model appears to be inappropriate, as mutants experience a higher rate of degradation. Biological examples of non-neutral behavior include the observation that the PINK1/Parkin pathway can select against deleterious mtDNA mutations *in vitro* (Suen *et al.* 2010) and *in vivo* (Kandul *et al.* 2016), as has repression of the mTOR pathway via treatment with rapamycin (Dai *et al.* 2013; Kandul *et al.* 2016). However, the necessity of performing a genetic/pharmacological intervention to clear mutations via this pathway suggests that the ability of tissues to selectively remove mitochondrial mutants under physiological conditions is weak. Consequently, neutral models such as our own are useful in understanding how the distribution of heteroplasmy evolves through time under physiological conditions. Indeed, it has been recently shown that mitophagy is basal (McWilliams *et al.* 2016) and can proceed independently of PINK1 *in vivo* (McWilliams *et al.* 2018), perhaps suggesting that mitophagy has nonselective aspects—although this is yet to be verified conclusively.

We have paid particular attention to the case of postmitotic tissues, since these tissues are important for understanding the role of mitochondrial mutations in healthy aging (Khrapko and Vijg 2009; Kauppila *et al.* 2017). A typical rate of increase of heteroplasmy variance predicted by Equation 13 given our nominal parameterization (Table S2) is $V\prime \left(h\right)/t=V\left(h\right)/\left(E\left(h\right)\left(1-E\left(h\right)\right)t\right)=2\mu f_{s}/n\approx 2.3\times 10^{-5}$ day^−1^ $\left(f_{s}=0.5,n=1000\right)$. This value accounts for the accumulation of heteroplasmy variance which is attributable to turnover of the mitochondrial population in a postmitotic cell. However, in the most general case, cell division is also able to induce substantial heteroplasmy variance. For example, V′(h)/t has been measured in model organism germlines to be ∼$9\times 10^{-4}$ day^−1^ in *Drosophila* (Solignac *et al.* 1987; Johnston and Jones 2016), $9\times 10^{-4}$ day^−1^ in NZB/BALB mice (Wai *et al.* 2008; Wonnapinij *et al.* 2008; Johnston and Jones 2016), and $2\times 10^{-4}$ day^−1^ in single Lehsten (LE) and Hohenberg (HB) mouse oocytes (Burgstaller *et al.* 2018). We see that these rates of increase in heteroplasmy variance are approximately an order of magnitude larger than predictions from our model of purely quiescent turnover, given our nominal parameterization. While larger mitophagy rates may also potentially induce larger values for V′(h)/t (see Poovathingal *et al.* 2012, and Figure S5C, corrsponding to $V\prime \left(h\right)/t\approx 3.5\times 10^{-4}$ day^−1^) it is clear that partitioning noise [or “vegetative segregation” (Stewart and Chinnery 2015)] is also an important source of variance in heteroplasmy dynamics (Johnston *et al.* 2015). Quantification of heteroplasmy variance in quiescent tissues remains an underexplored area, despite its importance in understanding healthy aging (Kauppila *et al.* 2017; Aryaman *et al.* 2019).

Our findings reveal some apparent differences with previous studies which link mitochondrial genetics with network dynamics (see Table S4). First, Tam *et al.* (2013, 2015) found that slower fission–fusion dynamics resulted in larger increases in heteroplasmy variance with time, in contrast to Equation 13 which only depends on fragmentation state and not absolute network rates. The simulation approach of Tam *et al.* (2013, 2015) allowed for mitophagy to act on whole mitochondria, where mitochondria consist of multiple mtDNAs. Faster fission–fusion dynamics tended to form heteroplasmic mitochondria, whereas slower dynamics formed homoplasmic mitochondria. It is intuitive that mitophagy of a homoplasmic mitochondrion induces a larger shift in heteroplasmy than mitophagy of a single mtDNA, hence slower network dynamics form more homoplasmic mitochondria. However, this apparent difference with our findings can naturally be resolved if we consider the regions in parameter space where the fission–fusion rate is much larger than the mitophagy rate, as is empirically observed to be the case (Cagalinec *et al.* 2013; Burgstaller *et al.* 2014a). If the fission–fusion rates are sufficiently large to ensure heteroplasmic mitochondria, then further increasing the fission–fusion rate is unlikely to have an impact on heteroplasmy dynamics. Hence, this finding is potentially compatible with our study, although future experimental studies investigating intramitochondrial heteroplasmy would help constrain these models. Tam *et al.* (2015) also found that fast fission–fusion rates could induce an increase in mean heteroplasmy, in contrast to Figure 2D which shows that mean heteroplasmy is constant with time. We may speculate that the key difference between our treatment and that of Tam *et al.* (2013, 2015) is the inclusion of cellular subcompartments which induces spatial effects which we do not consider here. The uncertainty in accounting for the phenomena observed in such complex models highlights the virtues of a simplified approach which may yield interpretable laws and principles through analytic treatment.

The study of Mouli *et al.* (2009) suggested that, in the context of selective fusion, higher fusion rates are optimal. This initially seems to contrast with our finding which states that intermediate fusion rates are optimal for the clearance of mutants (Figure 4A). However, the high fusion rates in that study do not correspond directly to the highly fused state in our study. Fission automatically follows fusion in Mouli *et al.* (2009), ensuring at least partial fragmentation, and the high fusion rates for which they identify optimal clearing are an order of magnitude lower than the highest fusion rate they consider. In the case of complete fusion, mitophagy cannot occur in the model of Mouli *et al.* (2009), so there is no mechanism to remove dysfunctional mitochondria. It is perhaps more accurate to interpret the observations of Mouli *et al.* (2009) as implying that selective fusion shifts the optimal fusion rate higher, when compared to the case of selective mitophagy alone. Therefore, the study of Mouli *et al.* (2009) is compatible with Figure 4A. Furthermore, Mouli *et al.* (2009) also found that when fusion is nonselective and mitophagy is selective, intermediate fusion rates are optimal, whereas Figure 4B shows that complete fragmentation is optimal for clearance of mutants. Optimality of intermediate fusion in the context of selective mitophagy in the model of Mouli *et al.* (2009) likely stems from two aspects of their model: (1) mitochondria consist of several units which may or may not be functional, and (2) the sigmoidal relationship between number of functional units per mitochondrion and mitochondrial “activity” (the metric by which optimality is measured). Points (1) and (2) imply that small numbers of dysfunctional mitochondrial units have very little impact on mitochondrial activity, so fusion may boost total mitochondrial activity in the context of small amounts of mutation. So while Figure 4B remains plausible in light of the study of Mouli *et al.* (2009) if reduction of mean heteroplasmy is the objective of the cell, it is also plausible that nonlinearities in mitochondrial output under cellular fusion (Hoitzing *et al.* 2015) result in intermediate fusion being optimal in terms of energy output in the context of nonselective fusion and selective mitophagy. Future experimental studies quantifying the importance of selective mitophagy under physiological conditions would be beneficial for understanding heteroplasmy variance dynamics. The ubiquity of heteroplasmy (Payne *et al.* 2012; Ye *et al.* 2014; Morris *et al.* 2017) suggests that a neutral-drift approach to mitochondrial genetics may be justified, which contrasts with the studies of Tam *et al.* (2013, 2015) and Mouli *et al.* (2009) which focus purely on the selective effects of mitochondrial networks.

To fully test our model, further single-cell longitudinal studies are required. For instance, the study by Burgstaller *et al.* (2018) found a linear increase in heteroplasmy variance through time in single oocytes. Our work here has shown that measurement of the network state, as well as turnover and copy number, are required to account for the rate of increase in heteroplasmy variance. Joint longitudinal measurements of $f_{s}$, *μ*, and *n*, with heteroplasmy quantification, would allow verification of Equation 13 and aid in determining the extent to which neutral genetic models are explanatory. This could be achieved, for instance, using the mito-QC mouse (McWilliams *et al.* 2016) which allows visualization of mitophagy and mitochondrial architecture *in vivo*. Measurement of $f_{s}$, *μ*, and *n*, followed by, *e.g.*, destructive, single-cell, whole-genome sequencing of mtDNA would allow validation of how *μ*, *n*, and $f_{s}$ influence V(h) and the rate of *de novo* mutation (see Figure 3). One difficulty is sequencing errors induced through, *e.g.*, PCR, which hampers our ability to accurately measure mtDNA mutation within highly heterogeneous samples (Woods *et al.* 2018). Morris *et al.* (2017) have suggested that single cells are highly heterogeneous in mtDNA mutation, with each mitochondrion possessing 3.9 single-nucleotide variants on average. Error-correction strategies during sequencing may pave the way toward high-accuracy mtDNA sequencing (Salk *et al.* 2018; Woods *et al.* 2018), and allow us to better constrain models of heteroplasmy dynamics.
